# Feasibility of using a pocket-sized ultrasound imaging device for detecting insulin-derived amyloidosis-like findings in patients with diabetes mellitus: a preliminary study

**DOI:** 10.20407/fmj.2025-028

**Published:** 2026-02-28

**Authors:** Naoko Kageura, Hiroji Takai, Yasuo Takatsu, Atsushi Suzuki, Shigeki Kobayashi, Kazuki Takano, Ayano Nakai, Junko Sugama, Kimie Takehara

**Affiliations:** 1 Graduate School of Health Sciences, Fujita Health University, Toyoake, Aichi, Japan; 2 Department of Radiology, Fujita Health University Hospital, Toyoake, Aichi, Japan; 3 Department of Medical Science and Molecular Imaging, Clinical Education Collaboration Unit, School of Medical Science, Fujita Health University, Toyoake, Aichi, Japan; 4 Department of Endocrinology, Metabolism and Diabetes, School of Medicine, Fujita Health University, Toyoake, Aichi, Japan; 5 Department of Radiological Technology, Clinical Education Collaboration Unit, School of Medical Science, Fujita Health University, Toyoake, Aichi, Japan; 6 Faculty of Nursing, School of Health Sciences, Fujita Health University, Toyoake, Aichi, Japan; 7 Center for Nursing Research and Social Implementation, Innovation Promotion Division, Research Promotion Headquarters, Fujita Health University, Toyoake, Aichi, Japan

**Keywords:** Insulin-derived amyloidosis, Pocket-sized ultrasound, Magnetic resonance imaging, Insulin injection, High-performance ultrasound

## Abstract

**Objectives:**

Insulin-derived amyloidosis (IDA) reduces insulin absorption, increasing the risk of poor glycemic control; however, early detection remains challenging. Although ultrasound can identify IDA, the effectiveness of portable pocket-sized devices has not been evaluated. This study was performed to evaluate the detectability of findings suggestive of IDA using a pocket-sized ultrasound device on the abdominal region, the most common site for stable insulin absorption in patients with diabetes mellitus.

**Methods:**

This cross-sectional observational study was conducted in the diabetes ward of a university hospital between July and December 2024. The participants were inpatients with diabetes who had been receiving insulin injections for more than 1 month. Findings suggestive of IDA were assessed through visual inspection and palpation, magnetic resonance imaging (MRI), high-performance ultrasonography, and a pocket-sized ultrasound device. The concordance rate between the pocket-sized ultrasound device and other assessment methods was calculated.

**Results:**

Of the 20 participants enrolled, 5 met the exclusion criteria; thus, data from 15 participants were included in the final analysis. Findings suggestive of IDA were identified in nine patients (60.0%) by visual inspection and palpation and in eight patients (53.3%) by the pocket-sized ultrasound device, high-performance ultrasound, and MRI. The concordance rate among the pocket-sized ultrasound device, high-performance ultrasound, and MRI was 100%, while the concordance rate between visual inspection with palpation and the pocket-sized ultrasound device was 80%.

**Conclusions:**

These findings suggest that a pocket-sized ultrasound device has the potential to detect findings suggestive of IDA and may be suitable for use in clinical practice.

## Introduction

Diabetes mellitus is a metabolic syndrome primarily characterized by chronic hyperglycemia resulting from insufficient insulin action.^1^ The number of adults living with diabetes has now exceeded 800 million worldwide, representing a four-fold increase since 1990.^2^ Individuals with diabetes can lead normal lives if their blood glucose levels are well controlled through appropriate treatment and self-care. However, poor glycemic control can lead to a wide range of acute and chronic complications. These complications not only reduce patients’ quality of life but also worsen their prognosis.^1^ To prevent such complications, patients must actively engage in self-care behaviors, including lifestyle modifications, dietary therapy, exercise, and oral pharmacological treatment. However, the duration of poor glycemic control has been shown to significantly influence the progression of subsequent complications.^3^ Therefore, if blood glucose remains inadequately controlled despite ongoing treatment, initiation of insulin therapy is recommended.^1^

Insulin therapy compensates for insufficient insulin levels, particularly in cases where pancreatic insulin secretion is markedly reduced or glycemic control remains poor. Although this therapy is highly effective, it requires patients to administer daily subcutaneous injections. Among the various injection sites, the lower abdomen is the most commonly used.^4^ In recent years, as the number of patients with diabetes continues to rise, increasing attention has been paid to complications associated with insulin administration. One such complication is insulin-related skin disorders (hereafter referred to as skin complications).^5^ These occur owing to repeated subcutaneous insulin injections at the same site, leading to induration or nodule formation.^6–8^

There are two main types of insulin-related skin complications. The first is lipohypertrophy (LH), which occurs when insulin is repeatedly injected into the same area of subcutaneous tissue, leading to locally elevated insulin concentrations. This promotes lipogenesis and causes enlargement of adipocytes.^9,10^ LH is believed to develop because of insulin’s hypertrophic effect on fat cells.^11,12^ The second type is insulin-derived amyloidosis (IDA), also known as an “insulin ball,” which reportedly develops through amyloid deposition in the dermis resulting from fibrosis caused by repeated insulin injections.^13,14^ Large-scale prevalence data on IDA are currently lacking^13^ because histological examination with biopsy is required for definitive diagnosis; however, numerous case reports have been published.^15^

The rate of insulin absorption at LH sites is reduced by approximately 20% compared with that at normal sites,^16^ whereas at IDA sites, it can be reduced by approximately 60% compared with that in unaffected areas.^17–19^ Consequently, the greater reduction in insulin efficacy in patients with IDA than in patients with LH may significantly worsen glycemic control. Moreover, patients who maintain glycemic control by injecting into IDA sites are at risk of hypoglycemia if injections are moved to unaffected areas.^20,21^ Therefore, prompt detection of IDA is crucial.

IDA lesion size is known to be irreversible—once an IDA enlarges, it does not regress even if insulin injections are discontinued.^17^ Therefore, early detection and prevention at the initial stage of formation are crucial. In clinical settings, nurses are primarily responsible for instructing patients on insulin injection techniques and self-injection practices. Assessment of insulin-related skin complications and selection of injection sites are generally performed through visual inspection and palpation. When swelling is observed at the injection site or a mass is detected by palpation, the likelihood of insulin-related skin complications is considered high. However, because early-stage IDA lesions are small, detecting them through visual inspection and palpation alone is difficult. By the time they are noticed, insulin efficacy may have already declined. One study revealed that among patients in whom no abnormalities were detected by visual inspection or palpation, ultrasound (US) identified LH in 19.9% of cases,^22^ suggesting that IDA may also be overlooked. IDA can reportedly be detected not only by visual and tactile examination but also by US and magnetic resonance imaging (MRI).^11,20,23–25^ Nevertheless, because biopsy is required for a definitive diagnosis, these imaging modalities cannot be considered conclusive for IDA detection. MRI is a large-scale procedure and not easily accessible for routine clinical use, whereas US is more practical, requiring no special preparation and being easier to perform.

Recent advances in ultrasonographic technology have led to the development of portable handheld US devices—often referred to as pocket-sized US devices—which are compact and increasingly used for nursing assessments of pressure injuries, edema, peripheral venous catheter insertion, and swallowing evaluations, demonstrating their growing clinical utility.^26^ If nurses in outpatient clinics or at the bedside could use pocket-sized US devices to assess insulin injection sites, they might detect IDA more reliably and at an earlier stage, enabling them to provide appropriate guidance to patients receiving insulin injections.

However, no previous studies have investigated the use of pocket-sized US devices for IDA detection. Because conventional stationary US systems offer higher resolution than pocket-sized devices, the ability of the latter to detect IDA remains uncertain. Therefore, this study was performed to evaluate the detection capability of a pocket-sized US device for identifying IDA, compared with that of MRI and a high-performance stationary US system. This preliminary study involved a small number of participants. The abdominal region was selected as the injection site because it is the most commonly used and allows stable insulin absorption.

## Methods

### Study design and setting

This single-center, cross-sectional observational study was conducted from July 2024 to December 2024 in the Department of Endocrinology, Metabolism, and Diabetes ward (hereafter referred to as the specialized ward) of our university hospital, as well as in other affiliated facilities of the university. The detailed inclusion and exclusion criteria for participants are described below.

### Participants

The inclusion criteria were a diagnosis of diabetes mellitus, age of 18 years or older, admission to the specialized ward at our university hospital, current self-administration of insulin injections into the abdominal region at least once daily, and continuation of this practice for more than 1 month.

The exclusion criteria were injection of insulin at sites other than the abdomen, impaired cognitive function or speech disorders that hindered effective communication, and inability to undergo MRI examinations because of metallic implants or other contraindications.

### Study variables

Because biopsy was not performed to avoid invasiveness, the IDA evaluated in this study will hereafter be referred to as “findings suggestive of IDA” to ensure accuracy.

#### 1. Visual inspection and palpation

Visual inspection was conducted with participants in the supine position, focusing on insulin injection sites to assess skin swelling, subcutaneous induration or masses, and pigmentation. Subcutaneous induration was defined as a localized hardening of the subcutaneous tissue caused by repeated needle punctures, presenting as a palpable firm area.^27^ A subcutaneous mass was defined as a palpable nodule within the subcutaneous tissue.^28^

Skin swelling, subcutaneous nodules, and pigmentation at the insulin injection sites were also examined. Palpation was performed in both the supine and standing positions, following the techniques described by Gentile et al.^29,30^ Two methods were used: (i) gentle, circular, and horizontal light palpation with the pads of the fingertips from the lower rib margin to the pubic area, and (ii) a pinch technique using the thumb and index finger to grasp the injection sites. These methods were employed to assess the presence and location of subcutaneous induration and nodules. Areas with hardened skin or subcutaneous fat tissue, as well as those showing swelling or nodules, were marked using a water-soluble pen. If no subcutaneous induration or nodules were observed, insulin injection sites were identified through patient interviews, after which the confirmed sites were marked with a water-based pen.

All assessments were performed by a researcher specializing in diabetes who held a nursing license. Participants were classified as having findings suggestive of IDA if skin elevations were observed during visual inspection or if subcutaneous abnormalities were detected on palpation. In cases where multiple findings were identified in a single participant, the following selection procedure was applied:

• When findings were observed on MRI, the most clearly defined MRI finding was chosen, and inspection and palpation were also conducted at the corresponding site.
• If no findings were detected on MRI but were evident on US, the most clearly defined US finding was selected, and inspection and palpation were performed at the same site.
• When findings were identified only through inspection and palpation, the most clearly define site based on those assessments was selected.

#### 2. MRI equipment

A 1.5T MRI system equipped with a 5-channel sensitivity encoding (SENSE) cardiac coil (Achieva; Philips Medical Systems, Best, The Netherlands) was used. The imaging protocol followed previous reports,^20,23,25^ employing T2-weighted imaging (T2WI) and fat-suppressed T2WI using spectral pre-saturation with inversion recovery (T2WI-SPIR). Both sequences used respiratory-triggered acquisition. The repetition times were set to 1,254 ms and 1,317 ms for T2WI and T2WI-SPIR, respectively. For both sequences, the echo time was 100 ms, the flip angle was 90°, and the fast spin-echo factor was 26. The field of view was 380 mm, with a slice thickness of 5 mm; 18 slices were acquired. Two signal averages were obtained. The acquisition matrix sizes were 344 for T2WI and 348 for T2WI-SPIR, with a reconstruction matrix size of 400 for both sequences. Parallel imaging was performed with a SENSE factor of 1.5. The imaging range extended from the inferior margin of the ribs to the pubic region. Participants were positioned supine with both upper limbs elevated near the head to avoid contact with the abdomen during scanning. All imaging procedures were performed by a medical radiological technologist collaborating in this study.

Imaging evaluation of findings suggestive of IDA was conducted by a radiologist. In this study, lesions showing low signal intensity on T2WI compared with adjacent subcutaneous fat and high signal intensity on fat-suppressed T2WI-SPIR were defined as findings suggestive of IDA.

If multiple findings were detected in a single participant, the most distinct MRI finding was selected when MRI abnormalities were present, and evaluations by visual inspection, palpation, and US were performed at the same site.

#### 3. US equipment

A high-performance US system (ALOKA ARIETTA850; FUJIFILM Corporation, Tokyo, Japan), hereafter referred to as high-performance US, was used with a linear probe operating at 18 MHz. A wireless US imaging device, iViz air (FUJIFILM Corporation, Tokyo, Japan), was used as the pocket-sized US device, equipped with a linear probe operating at a frequency range of 5–10 MHz.

US examinations were performed in the US room within the university hospital’s US Center. Imaging and US procedures were conducted by a researcher with a nursing license who had received training under the supervision of a certified sonographer specializing in superficial organs (hereafter referred to as the sonographer). The training covered the operation of both the high-performance and pocket-sized US devices. If multiple findings were detected in a single participant, the most clearly defined MRI finding was selected when available, and US evaluation was performed at the corresponding site. If no MRI findings were present but US demonstrated abnormalities, the most clearly defined US finding was selected, and visual inspection and palpation were performed at that same site.

US image evaluations were conducted according to the method described by Kikuchi et al.^24^ A lesion was classified as findings suggestive of IDA by the sonographer if both of the following criteria were met: an indistinct boundary was present between the dermis and subcutaneous fat, and the subcutaneous fat exhibited altered echogenicity compared with the surrounding tissue. Imaging sites were defined as areas where subcutaneous induration or nodules were identified through visual inspection or palpation. If no findings were observed, insulin injection sites were confirmed through patient interviews, marked with a water-soluble pen, and imaged using both the high-performance and pocket-sized US devices. When findings suggestive of IDA were detected on high-resolution US, an MRI skin marker developed by Takatsu et al.^31^ was applied as a landmark during MRI acquisition to ensure consistency of evaluation sites across visual inspection, palpation, US, and MRI.

#### 4. Basic characteristics

Data on age, sex, body mass index (kg/m^2^), type of diabetes, duration of insulin therapy (years), frequency of insulin injections, daily insulin dose, hemoglobin A1c (HbA1c) level, and type of insulin used were collected from electronic medical records.

### Research procedure

After obtaining informed consent, the participants were asked to expose their abdomen, after which a researcher with a nursing license performed visual inspection and palpation of the injection sites. Subsequently, the insulin injection sites were imaged in the US room of the University Hospital Ultrasound Center. MRI skin markers were applied to areas where changes in subcutaneous fat tissue were detected via US. Finally, MRI scans were conducted in the university’s MRI room. Before imaging, participants were screened with a metal detector in the preparation room to ensure they were not wearing any metallic objects, and they were then positioned supine for scanning.

### Independence of visual inspection, palpation, MRI, and US image evaluations

To ensure the independence of assessments, evaluations by visual inspection and palpation, MRI, and US were performed separately by different researchers. The evaluators did not share information regarding findings from visual inspection and palpation, MR scans, or US images, and no cross-checking of results was conducted among them.

### Statistical analysis

Descriptive statistics were used to summarize the participants’ basic characteristics. Concordance rates among assessments based on visual inspection and palpation, MRI findings, high-performance US findings, and pocket-sized US findings were evaluated. Concordance between modalities was defined as agreement on the presence or absence of findings at the same injection site according to the predefined criteria. The concordance rate was calculated as follows: (number of positive agreements + number of negative agreements)÷total number of sites×100%. All statistical analyses were performed using SPSS Statistics 26 for Windows (IBM Corp., Armonk, NY, USA).

### Ethics statement

This study was conducted in accordance with the ethical guidelines for medical research involving human participants established by the Ministry of Health, Labour and Welfare of Japan, and adhered to the principles of the Declaration of Helsinki.^32^ Written explanations were provided to all participants, who then gave written informed consent. The study protocol was approved by the Ethics Committee of our university (Approval No. HM24-021) on 25 April 2024.

## Results

Of the 20 participants enrolled in this study, 5 met the exclusion criteria. Consequently, 15 participants were included in the analysis.

### Basic characteristics

The participants’ basic characteristics are summarized in Table 1. Their median age was 57 years, median HbA1c level was 8.1%, median duration of insulin therapy was 20 years, and median daily insulin dose was 36 units (Table 1).

### Comparison of findings suggestive of IDA assessed by pocket-sized US device, high-resolution US, MRI, and visual inspection with palpation

**Figure 1:**
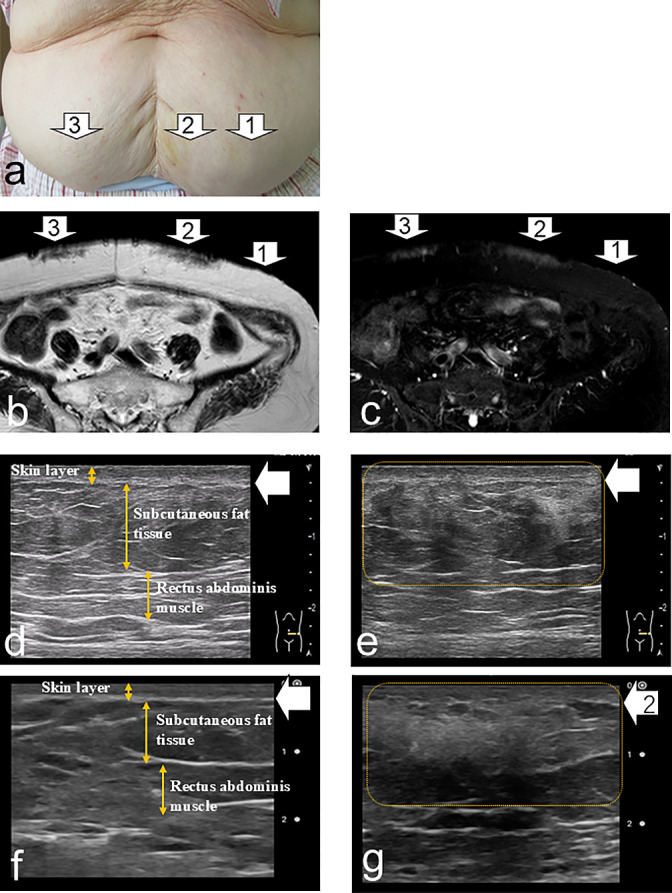
Subcutaneous induration and deep subcutaneous mass detected by visual inspection and palpation (Patient 12) a: No skin abnormalities were observed at a site with a low frequency of insulin injections (white arrow 1). Subcutaneous induration and a deep subcutaneous mass were identified at the insulin injection sites (white arrows 2 and 3). b: T2-weighted image of the lower left abdomen at a site with a low frequency of insulin injections (white arrow 1) showing no abnormalities. T2-weighted images of sites with subcutaneous induration and masses (white arrows 2 and 3) display low signal intensity in the areas indicated by the arrows compared with the surrounding subcutaneous fat tissue. c: Fat-suppressed T2-weighted image of the lower left abdomen at a site with a low frequency of insulin injections (white arrow 1) showing no abnormalities. Fat-suppressed T2-weighted images of sites with subcutaneous induration and masses (white arrows 2 and 3) demonstrate high signal intensity in the regions indicated by the arrows compared with the surrounding subcutaneous fat tissue. d: High-resolution ultrasound image of the lower left abdomen at a site with a low frequency of insulin injections (white arrow 1) showing a well-defined boundary between the dermis and subcutaneous fat. The dermis (white arrow) is clearly visualized, and the echogenicity of the subcutaneous fat is within normal limits. e: High-resolution ultrasound image of the lower left abdomen at a site with subcutaneous induration and mass (white arrow 2) showing an indistinct dermis–subcutaneous fat boundary (white arrow) and altered echogenicity of the subcutaneous fat compared with the surrounding tissue (yellow dotted line). f: Pocket-sized ultrasound image of the lower left abdomen at a site with a low frequency of insulin injections (white arrow 1) showing a well-defined dermis–subcutaneous fat boundary. The dermal layer (white arrow) is clearly visualized, and the echogenicity of the subcutaneous fat appears normal. g: Pocket-sized ultrasound image of the lower left abdomen at a site with subcutaneous induration and mass (white arrow 2) showing an indistinct dermis–subcutaneous fat boundary (white arrow) and altered echogenicity of the subcutaneous fat relative to the surrounding tissue (yellow dotted line).

**Figure 2:**
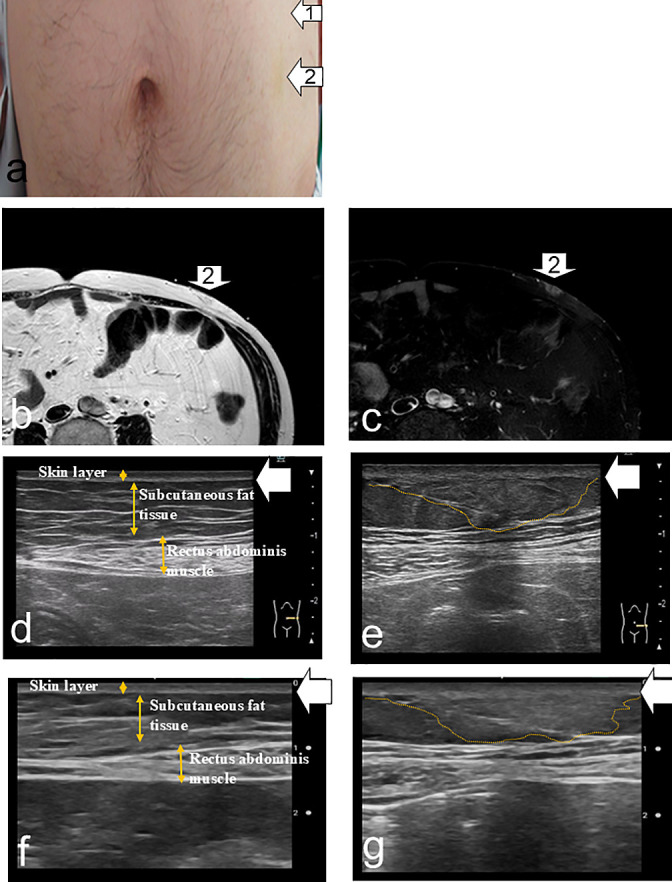
No subcutaneous induration or mass detected by visual inspection and palpation (Patient 3) a: Site with a low frequency of insulin injections (white arrow 1). Insulin injection site (white arrow 2). b: T2-weighted image of the insulin injection site (white arrow 2) showing low signal intensity in the region indicated by the arrow compared with the surrounding subcutaneous fat tissue. c: Fat-suppressed T2-weighted image of the insulin injection site (white arrow 2) demonstrating a hyperintense signal in the region indicated, relative to the surrounding subcutaneous fat tissue. d: High-resolution ultrasound image of the upper left abdomen at a site with a low frequency of insulin injections (white arrow 1) showing a well-defined dermis–subcutaneous fat boundary. The dermis (white arrow) is clearly visualized, and the subcutaneous fat exhibits normal echogenicity. e: High-resolution ultrasound image of the insulin injection site (white arrow 2) showing an indistinct dermis–subcutaneous fat boundary (white arrow) and altered echogenicity of the subcutaneous fat compared with the surrounding tissue (yellow dotted line). f: Pocket-sized ultrasound image of the upper left abdomen at a site with a low frequency of insulin injections (white arrow 1) showing a well-defined dermis–subcutaneous fat boundary. The dermis (white arrow) is clearly visualized, and the subcutaneous fat demonstrates normal echogenicity. g: Pocket-sized ultrasound image of the insulin injection site (white arrow 2) showing an indistinct dermis–subcutaneous fat interface (white arrow) and altered echogenicity of the subcutaneous fat relative to the surrounding tissue (yellow dotted line).

**Figure 3:**
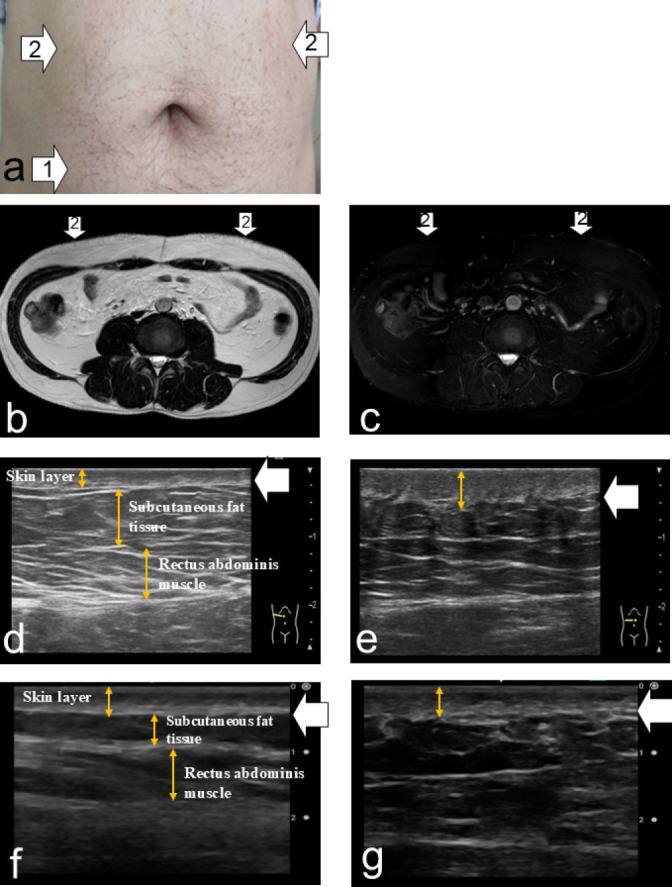
Skin swelling detected by visual inspection and palpation (Patient 13) a: Site with a low frequency of insulin injections (white arrow 1). Skin swelling was observed at the insulin injection sites (white arrows 2 and 3). b: No abnormalities were observed on T2-weighted images at the insulin injection sites (white arrows 2 and 3). c: No abnormalities were observed on fat-suppressed T2-weighted images at the insulin injection sites (white arrows 2 and 3). d: High-resolution ultrasound image of the lower right abdomen at a site with a low frequency of insulin injections (white arrow 1) showing a well-defined dermis–subcutaneous fat boundary. The dermis (white arrow) is clearly visualized, and the subcutaneous fat demonstrates normal echogenicity. e: High-resolution ultrasound image of the insulin injection site (white arrow 2) showing epidermal and dermal thickening, a clearly visualized dermis at the dermis–subcutaneous fat boundary (white arrow), and normal echogenicity of the subcutaneous fat. f: Pocket-sized ultrasound image of the lower right abdomen at a low-frequency insulin injection site (white arrow 1) showing a well-defined dermis–subcutaneous fat boundary. The dermis (white arrow) is clearly visualized, and the subcutaneous fat demonstrates normal echogenicity. g: Pocket-sized ultrasound image of the insulin injection site (white arrow 2) showing skin thickening involving the epidermis and dermis, a clearly visualized dermis at the dermis–subcutaneous fat boundary (white arrow), and normal echogenicity of the subcutaneous fat.

Findings suggestive of IDA were detected in eight patients (53.3%) by both the pocket-sized US device and the high-performance US, showing a 100% concordance rate with MRI (Figure 1, Table 2). Discrepancies between the pocket-sized US and visual inspection with palpation were observed in three patients (20.0%). In one patient who was positive on both MRI and US but negative by visual inspection and palpation (Patient 3), both the pocket-sized and high-performance US revealed indistinct boundaries between the dermis and subcutaneous fat, as well as altered echogenicity within the subcutaneous tissue. However, these changes were qualitatively diffuse and heterogeneous (Figure 2). Additionally, MRI showed that the size of the findings suggestive of IDA was considerably smaller than those identified in other MRI-positive cases. By contrast, in two patients who were negative on both MRI and US but positive by visual inspection or palpation (Patients 2 and 13), thickening of the epidermis and dermis was observed on both pocket-sized and high-performance US. However, the boundary between the dermis and subcutaneous fat was distinct, and echogenicity remained normal (Figure 3). No MRI findings suggestive of IDA were observed in these patients.

## Discussion

This preliminary study focused on patients with diabetes who administered insulin injections in the abdominal region and examined the feasibility of detecting findings suggestive of IDA using a pocket-sized US device. For the first time, we demonstrated a 100% concordance rate between pocket-sized US assessments and those obtained using MRI and high-performance US. These results suggest that a pocket-sized US device may be a practical tool for identifying IDA. However, the concordance rate between visual inspection with palpation and the pocket-sized US device was 80%, with discrepancies observed in three cases when visual inspection and palpation were used as the reference standards.

In this study, findings suggestive of IDA were observed in 60.0% of participants by visual inspection and palpation, and in 53.3% by MRI, high-performance US, and the pocket-sized US device. Both rates are higher than the 41.8% prevalence of LH reported in previous studies.^33^ A notable characteristic of this study’s participants was that 60% received four or more insulin injections per day, with a median daily insulin dose of 36 units and a relatively high median HbA1c level of 8.1%. One possible explanation for these findings is the inclusion of many patients admitted for preoperative glycemic control, which may have temporarily increased their insulin doses or injection frequency in preparation for surgery. Previous studies have reported a significant association between higher daily injection frequency or larger daily insulin doses and the presence of LH.^34^ Although these studies did not specifically examine IDA, similar factors may have contributed to IDA development in the present population.

Despite the findings of this study, discrepancies between visual inspection with palpation and US or MRI were observed in 20% of patients, a rate comparable to the discrepancy reported for LH detection in a previous study.^35^ In one patient who was positive on both MRI and US but negative by visual inspection and palpation, the lesion may not have reached a palpable hardness or size, making detection difficult. Therefore, the use of pocket-sized US devices by nurses for patients receiving insulin injections may allow earlier detection of findings suggestive of IDA that cannot be identified through visual inspection or palpation alone. This could lead to more accurate assessments for selecting appropriate injection sites and potentially reduce the number of patients who experience difficulty achieving glycemic control due to IDA formation.

To the best of our knowledge, this is the first study to demonstrate a 100% concordance rate for findings suggestive of IDA among pocket-sized US, MRI, and high-performance US. However, one limitation is that findings detected by the pocket-sized US device do not definitively confirm IDA. Therefore, in addition to conventional bedside or outpatient assessments of insulin injection sites using visual inspection and palpation, we propose incorporating pocket-sized US devices into evaluations of patients who show no abnormalities by these standard methods. This approach may enable earlier detection of findings suggestive of IDA that might otherwise be overlooked, leading to improved patient education on proper injection site selection. Consequently, it could help prevent new IDA formation, reduce unnecessary increases in insulin requirements, improve glycemic control, and ultimately mitigate the risk of diabetic complications.

As a result of this preliminary study, we found that transportation to the MRI room and the imaging process required considerable time, placing a slight burden on participants. Therefore, in future studies, it will be necessary to optimize the imaging procedure to shorten scan time. Other study variables were confirmed to be feasible without imposing any noticeable burden on participants.

### Limitations

This study has several limitations. First, the findings identified by the pocket-sized US device did not definitively confirm IDA. Thus, methods to validate these diagnoses are needed. Although biopsy is required to confirm amyloid deposition and establish a definitive diagnosis of IDA, it is highly invasive and impractical for large-scale studies. As an alternative, monitoring changes in blood glucose levels and HbA1c following changes in injection sites could serve as an indirect validation method. Second, differences in imaging capability were noted between the pocket-sized US device and the high-performance US system. These discrepancies may stem from variations in device resolution, frequency, and image processing functions, leading to differences in the depiction of fine subcutaneous fat structures (Figures 1–3). Third, regarding the clinical use of pocket-sized US devices by nurses, it will be essential to establish clear criteria for identifying findings suggestive of IDA. One possible approach in future research is to employ qualitative sketch techniques to visually describe the distinctive features of pocket-sized US images. Because this was a pretest, further large-scale, multicenter studies are warranted to confirm the validity and generalizability of our results.

## Conclusion

In this study, we evaluated the capability of a pocket-sized US device, which allows easy visualization of internal structures, to detect findings suggestive of IDA. The results demonstrated 100% concordance between findings identified by the pocket-sized US device and those detected by MRI and high-performance US. Therefore, pocket-sized US devices could serve as practical and effective tools for the early detection of findings suggestive of IDA by nurses at the bedside or in outpatient settings, thereby facilitating timely patient education and appropriate clinical intervention.

## Figures and Tables

**Table 1:** Patients’ basic characteristics (n=15)

Variable	Category	
Age, years		57 (33–84)
Sex	Male	7 (46.7)
	Female	8 (53.3)
Type of diabetes	Type 1	6 (40.0)
	Type 2	9 (60.0)
Body mass index, kg/m^2^		25.6 (19.0–33.2)
HbA1c, %		8.1 (7.3–11.7)
Reason for hospitalization	Preoperative glycemic control	9 (60.0)
	Evaluation of complications	6 (40.0)
Duration of insulin therapy, years		20 (1–46)
Insulin injection frequency	Once daily	3 (20.0)
	Twice daily	1 (6.7)
	Three times daily	2 (13.3)
	Four or more times daily	9 (60.0)
Daily insulin dose, units		36 (2–94)
Number of insulin formulations used	One	4 (26.6)
	Two	10 (66.7)
	Three	1 (6.7)
Insulin formulations used (multiple responses possible)	Rapid-acting (NovoRapid, Apidra, Humalog, Aspart BS)	10
	Premixed (Ryzodeg)	2
	Intermediate-acting (Humulin N)	1
	Long-acting (Tresiba, Levemir, Glargine BS, Lantus XR)	12

Data are presented as n (%), n, or median (range).

**Table 2:** Comparison of “findings suggestive of IDA” assessed using pocket-sized ultrasound, high-resolution ultrasound, MRI, and visual inspection with palpation

Patient ID	History of insulin use (years)	Insulin dose before admission (U/day)	Injection frequency (times/day)	HbA1c (%)	Type of insulin therapy	Ultrasound findings by device^a^		MRI findings^b^	Visual inspection^c^	Palpation^d^
						Pocket-sized ultrasound findings	High-performance ultrasound findings			
1	1	14	1	7.5	MDI					
2	23	94	4	10.6	MDI				+	
3	9	26	1	8.1	MDI	+	+	+		
4	4	2	1	10.9	MDI					
5	6	37	4	7.5	MDI					
6	36	32	2	10.2	MDI	+	+	+	+	+
7	27	29	4	9.4	MDI	+	+	+	+	+
8	20	52	4	8.1	MDI	+	+	+	+	+
9	30	26	4	7.3	MDI					
10	23	36	3	7.9	MDI	+	+	+	+	+
11	6	24	4	8.5	MDI	+	+	+	+	+
12	27	48	5	8.2	MDI	+	+	+	+	+
13	13	60	3	8.1	MDI				+	
14	46	40	4	8.0	MDI	+	+	+	+	+
15	12	36	4	11.7	MDI					

Note: Each positive finding (+) was evaluated based on the results of MRI, ultrasound, and inspection/palpation at the same site, using the most clearly defined MRI finding as the reference. ^a^ A finding was considered suggestive of IDA when both of the following criteria were met: the boundary between the dermis and subcutaneous fat tissue was indistinct, and the echogenicity of the subcutaneous fat tissue differed from that of the surrounding tissue. ^b^ A lesion showing lower signal intensity than subcutaneous fat on T2-weighted imaging and higher signal intensity than surrounding fat on fat-suppressed T2-weighted imaging was judged as a finding suggestive of IDA. ^c^ A case was considered positive if skin swelling, subcutaneous induration, or a subcutaneous mass was observed at any of the injection sites. ^d^ A case was considered positive when either subcutaneous induration or a subcutaneous mass was detected using either of the two palpation methods. Abbreviations: MDI, multiple daily injections; HbA1c, hemoglobin A1c; IDA, insulin-derived amyloidosis; MRI, magnetic resonance imaging; U, units
